# Mining immune-related genes with prognostic value in the tumor microenvironment of breast invasive ductal carcinoma

**DOI:** 10.1097/MD.0000000000025715

**Published:** 2021-04-30

**Authors:** Qiang He, Shuyin Xue, Qingbiao Wa, Mei He, Shuang Feng, Zhibing Chen, Wei Chen, Xinrong Luo

**Affiliations:** a Department of Cosmetic Plastic Surgery, Chengdu Second People's Hospital; b Smile Dental Clinic, Chengdu; c Department of Endocrine and Breast Surgery, The First Affiliated Hospital of Chongqing Medical University, Chongqing, China.

**Keywords:** breast invasive ductal carcinoma, immune score, stromal score, tumor microenvironment

## Abstract

The tumor microenvironment (TME) plays an important role in the development of breast cancer. Due to limitations in experimental conditions, the molecular mechanism of TME in breast cancer has not yet been elucidated. With the development of bioinformatics, the study of TME has become convenient and reliable.

Gene expression and clinical feature data were downloaded from The Cancer Genome Atlas database and the Molecular Taxonomy of Breast Cancer International Consortium database. Immune scores and stromal scores were calculated using the Estimation of Stromal and Immune Cells in Malignant Tumor Tissues Using Expression Data algorithm. The interaction of genes was examined with protein-protein interaction and co-expression analysis. The function of genes was analyzed by gene ontology enrichment analysis, Kyoto Encyclopedia of Genes and Genomes analysis and gene set enrichment analysis. The clinical significance of genes was assessed with Kaplan-Meier analysis and univariate/multivariate Cox regression analysis.

Our results showed that the immune scores and stromal scores of breast invasive ductal carcinoma (IDC) were significantly lower than those of invasive lobular carcinoma. The immune scores were significantly related to overall survival of breast IDC patients and both the immune and stromal scores were significantly related to clinical features of these patients. According to the level of immune/stromal scores, 179 common differentially expressed genes and 5 hub genes with prognostic value were identified. In addition, the clinical significance of the hub genes was validated with data from the molecular taxonomy of breast cancer international consortium database, and gene set enrichment analysis analysis showed that these hub genes were mainly enriched in signaling pathways of the immune system and breast cancer.

We identified five immune-related hub genes with prognostic value in the TME of breast IDC, which may partly determine the prognosis of breast cancer and provide some direction for development of targeted treatments in the future.

## 1 Introduction

Breast cancer is the most common malignant tumor in women worldwide.^[1]^ Although comprehensive treatments such as surgery, chemotherapy, radiotherapy and molecular targeted therapy have effectively reduced the mortality rate of breast cancer, a large number of patients still die from local recurrence and distant metastasis.^[2]^ Increasing evidence indicates that failures in treatment may be attributed to dysfunction in the tumor microenvironment (TME).^[3]^

The TME refers to the environment in which tumors occur, grow, invade and metastasize, which is divided into tumor components and non-tumor components.^[4]^ The latter contains cellular components (such as immune cells, fibroblasts, endothelial cells, inflammatory cells, adipocytes and other stromal cells) and non-cellular components (such as cytokines and extracellular matrix).^[5]^ The TME is in a dynamic state and is heterogeneous between different tumors.^[4]^ Stromal cells interact with tumor cells by secreting extracellular matrix proteins, chemokines and cytokines, resulting in activation of tumor cells that already have genetic abnormalities.^[6]^ Studies have shown that the clinical characteristics and prognoses of patients can be predicted by analyzing the features of gene expression and cell infiltration in the stromal environment.^[7–9]^ Tumor-infiltrating lymphocytes (TILs) are important prognostic indicators for triple-negative breast cancer (TNBC).^[10]^ Tomioka et al. found that low levels of TILs and high levels of programmed death-ligand 1 (PD-L1) in the TME are unfavorable to the prognosis of TNBC patients.^[11]^ In addition, blockade of some immune checkpoint proteins, such as CTLA-4, PD-1 and PD-L1, shows promising effects in breast cancer treatment.^[12,13]^ Therefore, the study of TME is of great significance for the early diagnosis and targeted treatment of breast cancer.

In recent years, with the development of high-throughput genome sequencing technology, many biological information databases of high-quality have emerged, such as The Cancer Genome Atlas (TCGA; https://portal.gdc.cancer.gov), Molecular Taxonomy of Breast Cancer International Consortium (METABRIC; http://www.cbioportal.org), Gene Expression Omnibus (GEO; https://www.ncbi.nlm.nih.gov/geo) and International Cancer Genome Consortium (https://icgc.org), which can be analyzed freely by researchers, providing the support of big data for tumor research. Yoshihara et al. designed the Estimation of Stromal and Immune Cells in Malignant Tumor Tissues Using Expression Data (ESTIMATE) algorithm based on genetic sequencing data of tumor samples to study the infiltration of immune cells and stromal cells in the TME and assess tumor purity.^[14]^ The ESTIMATE algorithm generates an immune score, stromal score and estimate score through GSEA, which represent the infiltration of immune cells, the presence of stroma and tumor purity of the TME, respectively.^[14]^ At present, the ESTIMATE algorithm has been used to analyze the TME of various tumors, such as breast cancer, glioblastoma and prostate cancer.^[15–17]^ Studies on breast cancer have found that immune and stromal scores are related to the clinical characteristics and prognoses of patients, and that scores differ between races.^[18]^ However, the detailed characteristics and molecular mechanisms of TME in breast cancer have not yet been fully elucidated.

In this study, the ESTIMATE algorithm was utilized to study the relationship between immune/stromal scores and clinical characteristics of breast invasive ductal carcinoma (IDC) patients. The R project was used to screen for hub genes with clinical significance in the TME of IDC, which may provide clues about the underlying molecular mechanisms active in breast TME.

## 2 Methods

### 2.1 Database

All data in this study are from public domain, no approval from the ethics committee is required. Gene expression profiles and clinical data of breast cancer patients were downloaded from TCGA (https://portal.gdc.cancer.gov) and the METABRIC database (http://www.cbioportal.org). Immune scores and stromal scores were calculated using the ESTIMATE algorithm in the R project (www.r-project.org).^[14]^ The immune/stromal scores and gene expression levels were compared between groups according to age, molecular subtype, TNM stage, adjuvant treatment, cellularity and menopause state.

### 2.2 Identification of differentially expressed genes (DEGs)

DEGs were selected using R package “limma” with FDR < 0.05 and log2 FC> 1.^[19]^ The heatmap was drawn with R package “pheatmap”,^[20]^ and the Venn diagram was drawn with R package “VennDiagram”.^[21]^

### 2.3 Functional and pathway enrichment analysis of DEGs

Functional and pathway enrichment analysis of DEGs was performed using R packages “clusterProfiler”, “org.Hs.eg.db,” “enrichplot” and “ggplot2”.^[22]^ The gene ontology analysis included biological process (BP), molecular function (MF) and cell component (CC). Kyoto Encyclopedia of Genes and Genomes analysis shows the enrichment of DEGs in signaling pathways.^[23]^ Gene set enrichment analysis (GSEA) (https://gsea-msigdb.org) was performed to study the enrichment in signaling pathways of the hub genes with data from the METABRIC database.^[24]^

### 2.4 Protein-protein interaction (PPI) network analysis

The PPI network analysis of DEGs was performed with STRING (https://string-db.org).^[25]^ The minimum required interaction score was 0.7 (high confidence). Edges and nodes of the PPI were counted in screening hub genes. Co-expression analysis was performed to identify the genes most positively or negatively related to the hub genes.^[26]^

### 2.5 Prognostic analysis

Patients were divided into low and high expression groups according to the median expression level of genes and Kaplan-Meier survival analysis was used to analyze overall survival (OS) using the R package “survival”.^[27]^ The univariate and multivariate Cox regression analyses were performed to select genes with prognostic value and ROC analysis was performed to evaluate the diagnostic capability of genes with the package “survivalROC”.^[27]^

### 2.6 Statistical analysis

All statistical analyses were performed using R project (version 3.5) and SPSS 22.0 software (IBM, Armonk, NY, USA). *P* values were calculated with the log-rank test and *P* < 0.05 was considered statistically significant.

## 3 Results

### 3.1 Immune scores and/or stromal scores correlate with overall survival and clinical characteristics of breast IDC patients

**Figure 1:**
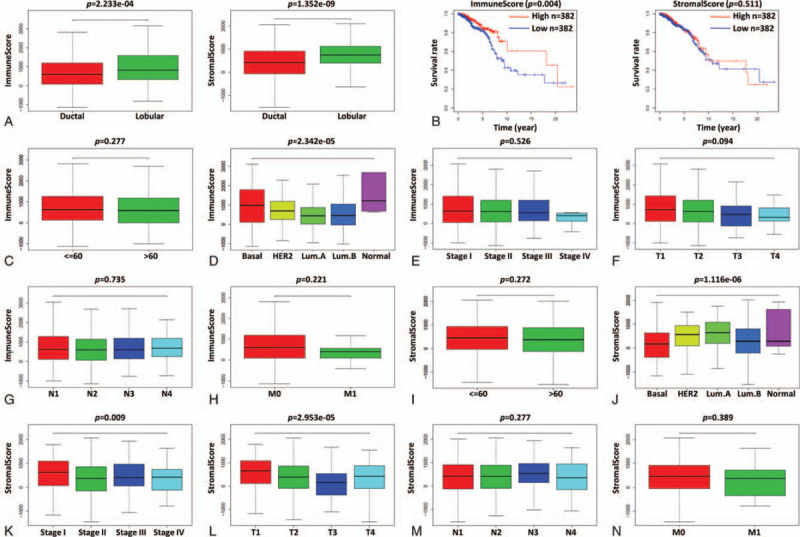
Immune scores and stromal scores correlate with clinical features and OS of breast IDC. (A) Comparison of immune/stromal scores between IDC and ILC of breast. (B) Relationship between immune/stromal scores and OS of patients with breast IDC. (C-H) Comparison of immune scores according to age, molecular subtype, AJCC stage and TNM stage of patients with breast IDC. (I-N) Comparison of stromal scores according to age, molecular subtype, AJCC stage and TNM stage of patients with breast IDC.

Immune scores and stromal scores, calculated with the ESTIMATE algorithm based on genetic sequencing data of tumor samples, reflect the presence of immune cells and stromal cells, respectively, and tumor purity. Studies have shown that the immune score and stromal score are valuable for tumor diagnosis and prognostic evaluation, including breast cancer.^[15–17]^ IDC and invasive lobular carcinoma (ILC) are the two most common pathological types of breast cancer.^[28]^ To compare the immune/stromal scores in these two types of breast cancer, the gene expression profiles of 964 breast cancer patients from TCGA were first downloaded, including 764 IDC and 200 ILC profiles. Then the immune scores and stromal scores were calculated by using the ESTIMATE algorithm. The results showed that the immune scores of IDC ranged from -1129.95 to 3661.56, with an average of 712.72, and the immune scores of ILC ranged from −796.34 to 3142.77, with an average of 941.17. The stromal scores of IDC ranged from −2070.44 to 2058.27, with an average of 401.84, and the stromal scores of ILC ranged from −1143.49 to 2099.46, with an average of 715.75. Therefore, both the immune scores and stromal scores of IDC were significantly lower than those of ILC (Fig. 1A, *P* < .0001).

Studies have shown that immune and stromal scores are related to clinical characteristics of tumors, such as age at diagnosis, clinical stage, tumor size, local and distant metastasis, pathological type, etc.^[14]^ In order to study the relationship between immune/stromal scores and clinical characteristics of IDC, the clinical data of the above 764 IDC patients were downloaded from TCGA, including age, AJCC stage, TMN stage, molecular subtypes and survival information (Table 1). The patients were divided into low and high immune/stromal score groups according to the median of scores, and Kaplan-Meier analysis was performed to compare the OS between the low score and high score groups. The results showed that the OS of patients with high immune scores was significantly longer than that of patients with low immune scores (Fig. 1B, p = 0.004). Next, the correlation between immune/stromal scores and clinical characteristics of IDC patients was analyzed. The results indicated that the immune scores were significantly related to the molecular subtype of breast IDC (Fig. 1D, *P* < .0001), and the stromal scores were significantly related to the molecular subtype (Fig. 1J, *P* < .0001), AJCC stage (Fig. 1K, *P* < .0001) and T stage (Fig. 1L, *P* = .009) of breast IDC. Other results were not statistically significant (Fig. 1C, E-I, M, and N). Therefore, immune scores and/or stromal scores are closely related to the clinical characteristics and OS of patients with IDC.

**Table 1:** Clinical characteristics and immune/stromal scores of patients from The Cancer Genome Atlas database with breast invasive ductal carcinoma.

Characteristics	Number of case (%) 764	Immune score (range)	Stromal score (range)
Age			
≤55	343 (44.9)	−983.81 to 3115.37	−1437.84 to 2058.27
>55	421 (55.1)	−1129.95 to 3661.56	−2070.44 to 2015.35
AJCC stage			
I	139 (18.2)	−1002.17 to 3060.80	−1166.48 to 1784.36
II	441 (57.7)	−1129.95 to 3661.56	−2070.44 to 2058.27
III	152 (19.9)	−763.283 to 3115.37	−1524.09 to 1933.37
IV	16 (2.1)	−735.324 to 1728.34	653.169 to 718.05
Unknow	16 (2.1)	−151.38 to 2170.75	−609.76 to 1610.23
T stage			
T1	217 (28.4)	−1018.74 to 3060.80	−1173.81 to 1784.36
T2	456 (59.7)	−1129.95 to 3661.56	−2070.44 to 2058.27
T3	58 (7.6)	−735.32 to 3115.37	−1093.36 to 1664.96
T4	31 (4.1)	−554.44 to 2060.92	−1524.09 to 1537.87
Unknow	2 (0.2)	−353.38 to 117.33	−396.75 to -244.47
N stage			
N0	350 (45.8)	−1018.74 to 3661.56	−2070.44 to 2015.35
N1	269 (35.2)	−1129.95 to 3115.37	−1268.12 to 2058.27
N2	93 (12.2)	−763.28 to 2800.07	−1328.32 to 1933.37
N3	38 (5.0)	−735.32 to 2136.87	−1056.02 to 1635.95
Unknow	14 (1.8)	−462.91 to 1441.60	−509.84 to 914.78
M stage			
M0	662 (86.6)	−1129.95 to 3661.56	−2070.44 to 2058.27
M1	18 (2.4)	−735.32 to 1728.34	−783.53 to 1627.82
Unknow	84 (11.0)	−870.44 to 2805.71	−1328.32 to 1699.96
Molecular subtype			
Luminal A	181 (23.7)	−943.65 to 3076.70	−1268.12 to 1754.23
Luminal B	141 (18.5)	−1018.74 to 3661.56	−2070.44 to 2015.35
HER2 enriched	65 (8.5)	−840.66 to 2800.07	−1093.36 to 1500.36
Basal-like	118 (15.4)	−1129.95 to 3115.37	−1173.81 to 1910.33
Normal-like	5 (0.7)	635.97 to 2694.14	−259.59 to 1933.37
Unknow	254 (33.2)	−870.44 to 2805.70	−1437.84 to 2058.27

### 3.2 DEGs in the TME of breast IDC and their functions

**Figure 2:**
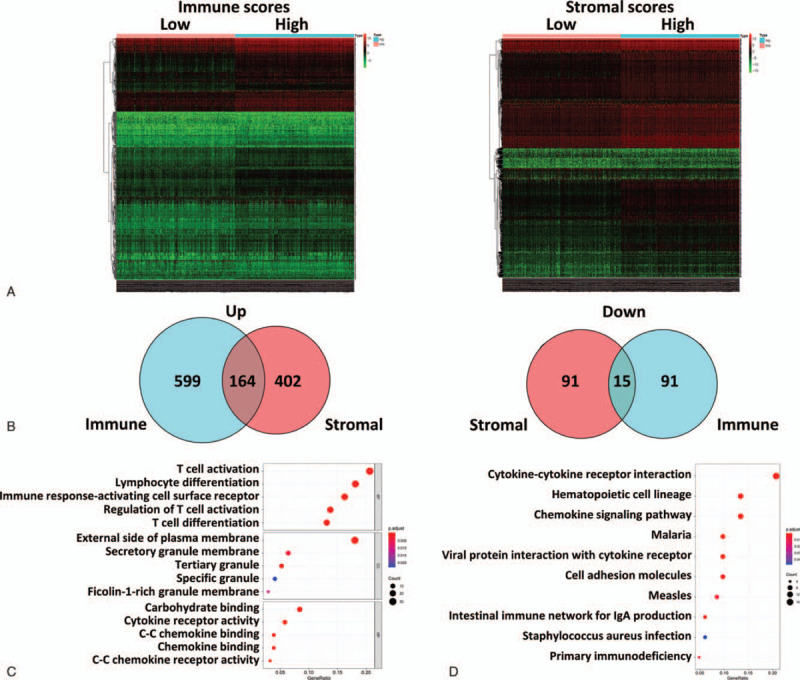
DEGs according to immune scores and stromal scores. The DEGs were compared between low and high immune/stromal score groups according to the median score. (A) Heatmap showing DEGs according to immune and stromal scores. (B) Venn diagram showing the intersection of DEGs between immune score and stromal score groups. (C) Results of GO functional enrichment analysis of the DEGs, including BP, MF and CC. (D) Top ten pathways from KEGG analysis of the DEGs.

Studies have shown that networks of gene regulation vary in different types of TMEs.^[29]^ To study the relationship between immune/stromal scores and gene expression, the gene expression profiles of 764 breast IDC patients were analyzed and the results are displayed in the form of a heatmap (Fig. 2A). The results showed that compared with the low immune score group, the high immune score group had 763 high expression genes and 106 low expression genes (fold change [FC]> 2, false discovery rate [FDR] < 0.05). Compared with the low stromal score group, the high stromal score group had 566 high expression genes and 106 low expression genes (FC> 2, FDR < 0.05). By intersecting these DEGs, we found that there were 164 common high-expressing genes and 15 common low-expressing genes (Fig. 2B).

To further analyze the function of the 179 common DEGs, Gene ontology function enrichment analysis was performed, including BP, MF and CC. The results showed that these genes were mainly enriched in functions of T cell activation, lymphocyte differentiation, immune response, plasma membrane and cytokine receptor activity. Figure 2C shows the top 15 enriched functions (Fig. 2C). We also performed Kyoto Encyclopedia of Genes and Genomes pathway analysis. These results showed that the 179 genes were mainly enriched in cytokine-cytokine receptor interaction, hematopoietic cell lineage and chemokine signaling pathways. Fig. 2D shows the top ten enriched pathways. Thus, these common DEGs are involved in the regulation of the immune process.

### 3.3 Hub genes in the TME of breast IDC

**Figure 3:**
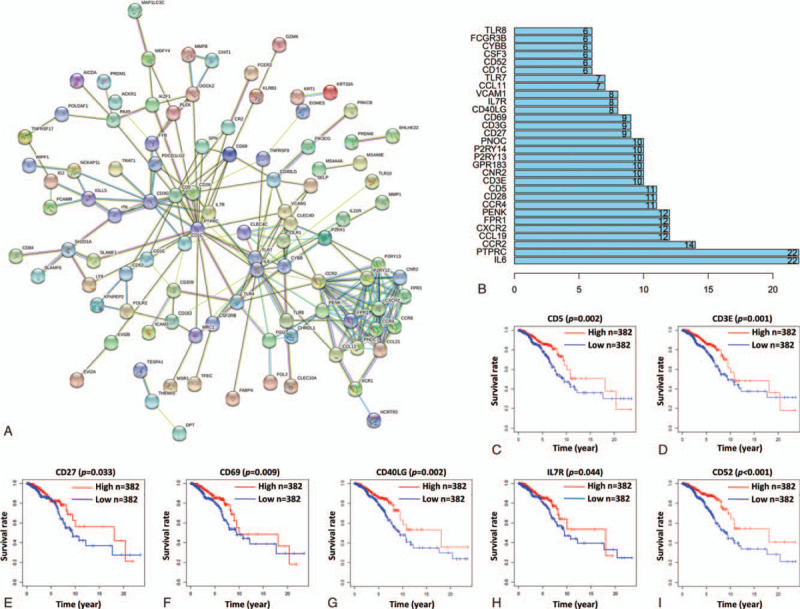
Hub genes in the TME of breast IDC. (A) PPI network of the DEGs. (B) Top 30 genes with the most nodes in the PPI network. (C-I) The Kaplan-Meier survival analysis of patients expressing CD5, CD3E, CD27, CD69, CD40LG, IL7R and CD52.

**Figure 4:**
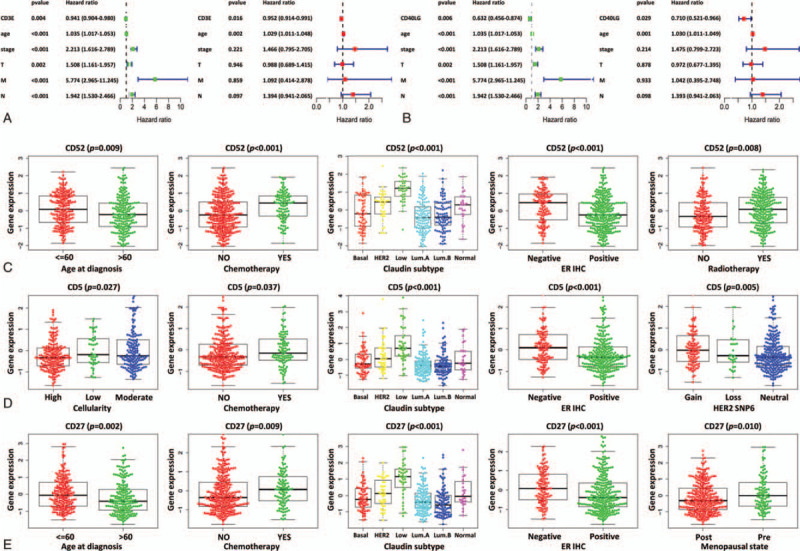
Clinical relevance of hub genes. (A-B) Univariate and multivariate Cox regression analyses of CD3E and CD40LG, respectively. (C-E) Relationship between gene expression levels and clinical characteristics of patients with breast IDC. C-E show CD52, CD5 and CD27, respectively.

To study the interaction of DEGs in the TME of breast IDC, a protein-protein interaction (PPI) network was constructed through the STRING database. The results showed that there were 223 edges and 101 nodes in the PPI network of these 179 common DEGs (Fig. 3A). The number of nodes of each gene were counted and the top 30 genes with the most nodes are displayed in Figure 3B. In order to identify the genes with clinical significance, Kaplan-Meier survival analysis was performed for these 30 genes in patients with breast IDC. The results showed that CD5, CD3E, CD40LG, CD52, CD27, CD69 and IL7R were significantly related to the OS of patients with breast IDC (Fig. 3C-I). Then univariate and multivariate Cox regression analysis of these seven genes was performed. The results indicated that the hazard ratio (HR) values of CD3E, CD5, CD27, CD40LG and CD52 were under 1 and statistically significant in both univariate and multivariate Cox regression analyses (Fig. 4A and B, Table 2). Thus, these five genes are likely the hub genes in the TME of breast IDC.

**Table 2:** Univariate and multivariate Cox regression analysis of patients from the cancer genome atlas database with breast invasive ductal carcinoma.

	OS			
	Univariate analysis		Multivariable analysis	
Variants	HR (95% CI)	*P*-value^∗^	HR (95% CI)	*P*-value
Age	1.035 (1.017–1.053)	<.001		
Stage	2.123 (1.616–2.789)	<.001		
T stage	1.508 (1.161–1.957)	.002		
N stage	1.942 (1.530–2.466)	<.001		
M stage	5.774 (2.965–11.245)	<.001		
CD3E	0.941 (0.904–0.980)	.004	0.952 (0.914–0.991)	.016
CD5	0.882 (0.801–0.971)	.010	0.910 (0.830–0.998)	.045
CD27	0.908 (0.847–0.972)	.006	0.927 (0.868–0.989)	.022
CD40LG	0.632 (0.456–0.874)	.006	0.710 (0.521–0.966)	.029
CD69	0.927 (0.845–1.018)	.113		
IL7R	0.952 (0.908–0.999)	.044		
CD52	0.978 (0.965–0.992)	.002	0.983 (0.969–0.997)	.014

### 3.4 Validation in the METABRIC database

To verify the clinical significance of these five hub genes, another large breast cancer database, METABRIC, was analyzed.^[30]^ The gene expression data and the complete clinical data of 384 patients with breast IDC from METABRIC were downloaded from the cBioPortal for Cancer Genomics database (http://www.cbioportal.org). The patients were divided into different groups according to their clinical features, such as age at diagnosis, cellularity, chemotherapy, Claudin subtype, ER status, and inferred menopausal status. Then the gene expression level between groups was compared, which showed that the expression levels of CD3E, CD5, CD27, CD40LG, and CD52 significantly differed between groups of clinical features (Fig. 4C-E, Table 3). Therefore, the expression levels of these five genes were related to clinical characteristics of breast IDC.

**Table 3:** Statistically significant clinical features of each hub gene from clinical correlation analysis.

Gene names	Statistically significant clinical features
CD3E	Cellularity, chemotherapy, Claudin subtype, ER status, inferred menopausal status
CD40LG	Cellularity, Claudin subtype
CD52	Age at diagnosis, chemotherapy, Claudin subtype, ER status, radiotherapy
CD5	Cellularity, chemotherapy, Claudin subtype, ER status, HER2 SNPs
CD27	Age at diagnosis, chemotherapy, Claudin subtype, ER status, inferred menopausal status

**Figure 5:**
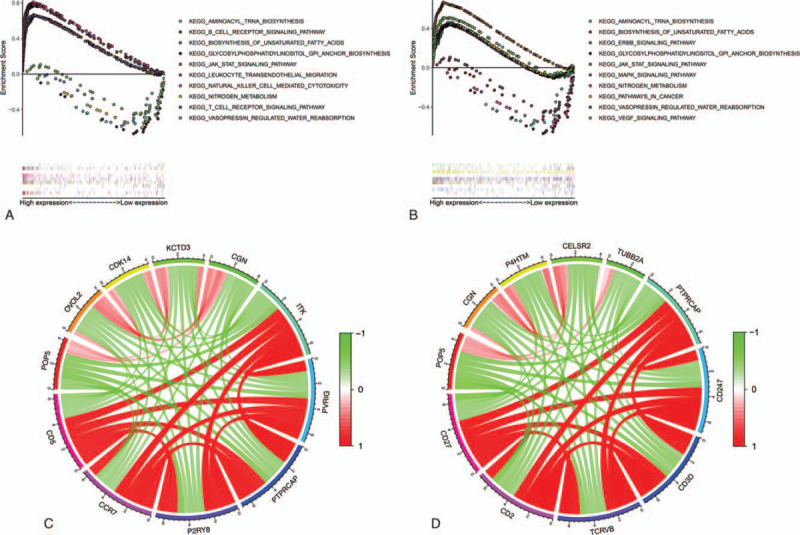
Enrichment analysis and co-expression analysis of hub genes. (A-B) GSEA of CD5 and CD27, respectively. C-D show co-expression analysis of CD5 and CD27, respectively.

To further study the function and protein-protein correlation of these five genes, GSEA and co-expression analysis were performed with data from METABRIC.^[24,26]^ The results of GSEA showed that the 5 genes were mainly enriched in immune-related and breast cancer-related signaling pathways, such as T and B cell receptor signaling pathways, natural killer cell-mediated cytotoxicity, MAPK, ERBB, WNT, VEGF signaling pathway, and others (Fig. 5A-B and Table 4). The results of co-expression analysis showed the genes with the strongest correlation with CD3E, CD5, CD27, CD40LG and CD52 (Fig. 5C-D and Table 5). Thus, we infer these five genes may play an important role in the TME to some extent.

**Table 4:** The most enriched signaling pathways of hub genes from gene set enrichment analysis.

KEGG pathways	Genes
KEGG_VEGF_SIGNALING_PATHWAY	CD3E/CD5/CD27/CD40LG/CD52
KEGG_JAK_STAT_SIGNALING_PATHWAY	CD3E/CD5/CD27/CD40LG/CD52
KEGG_MAPK_SIGNALING_PATHWAY	CD3E/CD27/CD40LG
KEGG_PATHWAYS_IN_CANCER	CD3E/CD5/CD27/CD40LG/CD52
KEGG_ERBB_SIGNALING_PATHWAY	CD5/CD27/CD40LG
KEGG_WNT_SIGNALING_PATHWAY	CD3E/CD27/CD40LG
KEGG_T_CELL_RECEPTOR_SIGNALING_PATHWAY	CD3E/CD5/CD27/CD40LG/CD52
KEGG_NATURAL_KILLER_CELL_MEDIATED_CYTOTOXICITY	CD3E/CD5/CD27/CD40LG/CD52
KEGG_LEUKOCYTE_TRANSENDOTHELIAL_MIGRATION	CD3E/CD5/CD27/CD40LG/CD52
KEGG_B_CELL_RECEPTOR_SIGNALING_PATHWAY	CD3E/CD5/CD27/CD40LG/CD52

**Table 5:** The top 10 related hub genes from co-expression analysis.

Genes	Positively related	Negatively related
CD3E	ITK/PTPN7/TBC1D10C/ACAP1/ZAP70	CDK14/POP5/TTC8/IFT81/CDKL3
CD5	CCR7/P2RY8/PTPRCAP/PVRIG/ITK	POP5/OVOL2/CDK14/KCTD3/CGN
CD27	CD2/TCRVB/CD3D/CD247/PTPRCAP	POP5/CGN/P4HTM/CELSR2/TUBB2A
CD40LG	GPR18/CCR7/TRAF3IP3/CD5/CXCR5	CDK14/CGN/OVOL2/KCTD3/DPCD
CD52	TCRVB/GZMA/GZMK/HCST/CD3D	P4HTM/CELSR2/POMT2/CCDC24/KDM4B

## 4 Discussion

In this study, the immune scores and stromal scores of breast IDC were calculated using data from TCGA, and the relationship between the scores and clinical characteristics was analyzed. Then, DEGs were analyzed according to the immune/stromal scores and the hub genes in the TME of breast IDC were identified with PPI analysis and univariate/multivariate Cox regression analysis. Finally, the clinical significance and the correlation with expression of these hub genes were validated with data from METABRIC.

Dysfunction of the TME is one of the most important causes of breast cancer recurrence and metastasis^[31]^. An increasing number of studies have recently focused on features of the breast TME. Chen et al. compared the characteristics of the TME of breast cancer in Asian and Western populations from GEO and TCGA databases by using ESTIMATE and CIBERSORT algorithms. Their results showed that the immune scores of Asian populations were higher than Western populations, especially for luminal B and HER2-enriched breast cancer patients.^[18]^ Vincent et al. found that the immune/stromal scores of breast cancer cell lines were inconsistent with those of the corresponding breast cancer tissue by using data from TCGA, GEO, UCSC Cancer Genomics Browser and cBio Cancer Genomics Portal databases.^[32]^ In a study of the relationship between DNA methylation and TME, Li et al. found that DNA methylation regulated the immune response by suppressing ACDY6 in the TME of luminal-like breast cancer.^[33]^ However, the detailed molecular mechanism for this effect in the TME of breast cancer has not yet been elucidated.

It has been reported that immune/stromal scores vary in different molecular subtypes and different histological types of breast cancer.^[34,35]^ In the present study, we compared the immune/stromal scores between breast IDC and ILC. The results showed that the immune scores of breast IDC were significantly lower than those of breast ILC, suggesting that different pathological types of breast cancer have different TME characteristics. According to the meaning of immune scores and stromal scores, we infer that this discrepancy is due to the difference in TME between IDC and ILC, that is, the immune and stromal components in the TME of IDC are less numerous than those of ILC. Several studies are consistent with our speculation. Tian et al. analyzed immune cell-type specific signatures of breast luminal (Lum) A IDC and ILC in TCGA and in the Genotype-Tissue Expression Project. Their results showed that LumA ILC had a higher proportion of high-immune phenotypes compared to LumA IDC. In addition, higher expression was observed for several critical immune checkpoint genes, such as CD274 (PD-L1), PDCD1 (PD-1) and CTLA4 in LumA ILC.^[36]^ Lee et al. collected 123 samples of invasive mammary carcinomas and observed the pattern of inflammation. They found that a combination of perilobular and perivascular inflammation composed of B and T cells was seen more frequently in ILC than in IDC.^[37]^ Zhao et al. performed a gene expression profiling study analyzing 21 ILC and 38 IDC samples. The results showed that stromal and adipose tissue markers, such as FBN1, KRT5, APM1 and FABP4, presented mainly in the ILC samples.^[38]^ However, there is one study that contradicts our speculation. Desmedt et al. compared the number of tumor-infiltrating lymphocytes (TILs) in ER-positive/HER2-negative ILC and IDC. The study indicated that the TIL levels were significantly lower in ILC compared with IDC.^[39]^ Thus, previous studies have explained our results in this study to some extent, but it is still too early to draw a conclusion and further research should be conducted to cover more subtypes of breast cancer and in a larger sample size. In addition, our results showed that LumA, LumB and HER2-enriched breast cancer have higher stromal scores and lower immune scores compared with basal-like breast cancer. Normal-like breast cancer had the highest immune scores (Fig. 1D), which is similar to previous findings.^[33,40]^

Current studies indicated that immune scores and stromal scores reflect the purity of the tumor and the level of immune response, that is, low immune/stromal scores indicate high tumor purity, relatively weak immune response and poor prognosis to some extent.^[14]^ In our study, we found that the stromal scores were significantly related to AJCC stage and T stage of breast IDC (as the stage increases, the stromal scores decrease). In addition, although not statistically significant, the immune/stromal scores were lower in patients younger than 60 years old (Fig. 5C and I) and in patients with distant metastasis (Fig. 5H and N). Therefore, the immune scores and stromal scores reflect the clinical characteristics of patients with breast IDC to a certain extent.

With PPI network analysis and univariate/multivariate Cox regression analysis, five hub genes in the TME of breast IDC with prognostic significance were identified: CD5, CD3E, CD40LG, CD52 and CD27. Receiver operating characteristic (ROC) curves were drawn with R package “survivalROC” to evaluate the diagnostic ability of these hub genes.^[27]^ However, none of the genes were statistically significant under the curve values (data not shown). Next, the clinical significance of these genes was validated in the METABRIC database. The results showed that the expression level of these five hub genes was related to the clinical features of patients with breast IDC and were enriched in immune-related and breast cancer-related signaling pathways. Finally, genes whose expression levels were most significantly related to these five hub genes were identified. Thus, our study identified five genes with prognostic significance from the TME of breast IDC.

The five hub genes, CD40LG, CD5, CD27, CD3E and CD52, are cluster of differentiation (CD) leukocyte surface antigens, which are mainly related to immune function.^[41]^Table 6 summarizes current studies on these five hub genes in breast cancer.^[42–59]^ CD40LG, also called CD154 or CD40L, is mainly expressed in activated T lymphocytes and belongs to the tumor necrosis factor family. It regulates the immune response by binding to its ligand CD40.^[60]^ Our results from PPI network analysis showed that CD40LG is closely related to many important proteins, including CD69, VCAM1 and TLR7, and its expression level is an independent prognostic factor in patients with breast IDC. Furthermore, the expression of CD40LG is closely related to cellularity and the Claudin subtype of breast IDC, and the results of GSEA indicated that CD40LG was mainly enriched in WNT, ERBB, VEGF, MAPK and JAK signaling pathways. We are mainly interested in CD40LG since several studies have already reported its function and molecular mechanism in the TME. Brummer et al. found that CCR2 signaling promoted breast cancer cell proliferation and invasion by inhibiting CD40LG while activating CCL2. In addition, high expression of CD40LG is a favorable indicator for the recurrence-free survival of patients with breast IDC.^[42]^ Mesenchymal stem cells edited with TNF-α and CD40L enhanced the anti-tumor immune function of mice by promoting the function of Th1 cells and inhibiting the function of Th2 and Treg cells.^[61]^ From immunotherapy research, CD8+ T cell-based adoptive cell therapy suppresses tumor development by activating dendritic cells and releasing tumor necrosis factor through the CD40/CD40LG signaling pathway.^[62]^ Soliman et al. found that a cell vaccine containing granulocyte macrophage-colony stimulating factor and CD40L enhanced anti-tumor ability in a mouse model of breast cancer by increasing infiltration of CD3+ lymphocytes through IL-2 and TNF-γ activation.^[63]^ Studies on non-solid tumors have shown that CD40LG and IL4 promote tumor cell proliferation and chemotherapy resistance by activating STAT3, NF-κB, ERK and AKT signaling pathways.^[64,65]^ Thus, CD40L plays an important role in the TME of various tumors, but the functions and molecular mechanism of CD40L in the TME of breast IDC requires further research.

**Table 6:** Previous studies on five hub genes in breast cancer.

Study	Breast cancer type	Gene	Brief summary
Brummer et al.^[42]^	Breast IDC	CD40LG	CCR2 signaling promoted breast cancer cell proliferation and invasion by inhibiting CD40LG while activating CCL2. In addition, high expression of CD40LG was a favorable indicator for recurrence-free survival of patients with breast IDC.
Tong et al.^[43]^	Breast cancer cell lines	CD40LG	Soluble recombinant CD40 ligand (CD40L) molecules effectively inhibited the growth of breast cancer *in vivo* by inducing apoptosis of breast cancer cells.
Gomes et al.^[44]^	Breast cancer cell lines	CD40LG	CD40LG inhibited the *in vitro* growth of CD40+ human breast cancer lines by blocking the cell cycle and inducing apoptosis of breast cancer cells.
Pan et al.^[45]^	Breast IDC	CD40LG	The expression levels of CD40/CD40L on B cells and T cells in breast IDC patients were significantly increased, and CD40/CD40L levels had a significant positive relationship with pathological grades.
Voorzanger-Rousselot et al.^[46]^	Breast cancer cell lines	CD40LG	CD40LG reduced the apoptosis of breast cancer cells induced by chemotherapeutic drugs.
Kim et al.^[47]^	Breast cancer cell lines	CD40LG	The CD40-CD40L interaction promoted the proliferation of breast cancer cells (MDA-MB-231) by increasing TGF-β production and Th17 differentiation.
Voorzanger-Rousselot et al.^[48]^	Breast cancer cell lines	CD40LG	CD40L was expressed on a CD40-positive breast cancer cell line (T47D) and induced an antiapoptotic signal when cells were exposed to cytotoxic agents.
Wang et al.^[49]^	Breast cancer cell lines	CD40LG	Co-expression of *Drosophila melanogaster* deoxyribonucleoside kinase and CD40L decreased cell proliferation and induced cell apoptosis of MDA-MB-231 and MCF7 cells *in vitro* and *in vivo*.
Yu et al.^[50]^	Breast cancer cell lines	CD40LG	CD4+T cells in cytokine-induced killer cells induced Fas-dependent apoptosis of MDA-MB-231 cells through CD40/CD40L ligation by inhibiting synthesis of c-FLIP.
Shousha et al.^[51]^	Breast IDC	CD5	Massive infiltration of axillary lymph nodes with CD5-positive B lymphocytes was found in a breast IDC patient. Strong staining for CD5 was also observed in tumor cells within the metastases of breast and lymph nodes.
Walsh et al.^[52]^	Breast cancer (pathological type not mentioned)	CD5	A negative correlation was found between CD5 positivity and tumor grade in breast cancer patients.
Alotaibi et al.^[53]^	Breast cancer cell lines	CD5	The use of a function-blocking anti-CD5 monoclonal antibody or knockout of CD5 inhibited tumor growth in a breast cancer mouse model by enhancing the capability of CD8+ T cells.
Xu et al.^[54]^	Breast cancer (pathological type not mentioned)	CD27	The rs3136550 CT and rs2267966 AT genotypes of CD27 were associated with a decreased risk of breast cancer. In haplotype analysis, the CCGAG haplotype conferred an increased risk of breast cancer. Significant associations were shown between the SNPs of CD27 and lymph node metastasis, and ER and PR status.
Han et al.^[55]^	Breast cancer cell lines	CD27	CD27 or 4–1BB-costimulated, self-enriched NKG2D CAR-redirected T cells effectively recognized and inhibited the proliferation of MDA-MB-231 cells *in vitro* and *in vivo*.
Wang et al.^[56]^	Breast cancer (pathological type not mentioned)	CD52	The expression level of CD52 was related to the prognosis and pathological stages of BRCA patients. Analysis based on RNA-seq and clinical data from TCGA datasets showed that CD52 was positively correlated with immune response-related pathways and immune metagenes.
Wang et al.^[57]^	Breast cancer (pathological type not mentioned)	CD52	The expression level of CD52 was related to the prognosis of breast cancer patients. Tumor-infiltrating immune cell analysis showed the relationship between CD52 expression and CD8+ T cells, activated memory CD4+ T cells, macrophage M1, and gamma delta T cells.
Khatibi et al.^[59]^	Breast cancer cell lines	CD3E	A recombinant anti-CD3E nanobody effectively suppressed angiogenesis and tumor cell proliferation in a breast cancer mouse model.
Moradi-Kalbolandi et al.^[58]^	Breast cancer cell lines	CD3E	A purified anti-CD3E nanobody effectively inhibited the growth of breast cancer *in vivo*.

CD5 is a glycoprotein receptor on the surface of T lymphocytes.^[66]^ A recent study found that the use of function-blocking anti-CD5 monoclonal antibody or the knockout of CD5 inhibits tumor growth in a breast cancer mouse model by enhancing the capability of CD8+ T cells.^[67]^ Hosaka et al. detected the expression of CD5 in both thymic cancer tissue and lymphoid stroma. Their results showed that the expression of CD5 was significantly increased at the junction of tumor and stroma, and its expression in lymphocytes was significantly higher than in tumor cells. Furthermore, high levels of CD5 indicated elevated Ki-67 and activated tumor cell proliferation.^[68]^ Another study on pancreatic ductal adenocarcinoma indicated that Bruton's tyrosine kinase promoted the proliferation of cancer cells by activating CD1d^hi^CD5^+^ regulatory B cells, which accumulated in the stroma of pancreatic lesions of mice.^[69]^

CD27 is a member of the tumor necrosis factor receptor superfamily, which regulates lymph cell activation and immunoglobulin synthesis by binding to its ligand CD70.^[70]^ Its potential in immune therapy has been recently noticed.^[71]^ Roberts et al. demonstrated that agonistic anti-CD27 antibodies inhibited the growth and metastasis of melanoma *in vivo* by enhancing the function of tumor-specific CD8+ T cells.^[72]^ In addition, clinical trials showed that an agonist of CD27, varlilumab, was effective and well tolerated in patients with advanced solid tumors by enhancing anti-tumor immunity driven by T cells.^[73]^

For CD52 and CD3E, there are relatively fewer studies on their activity in the TME. CD52, also known as CAMPATH-1 antigen, is a glycoprotein which presents on the surface of lymphocytes.^[74]^ Current research has mainly focused on the anti-tumor effects of the CD 52 antibody alemtuzumab in patients with lymphoma.^[75]^ Together with CD3-gamma, -delta and -zeta, CD3-epsilon/CD3E forms the T cell receptor-CD3 complex, which plays a key role in antigen recognition and signal transduction.^[76]^ Azadeh et al. found that a recombinant anti-CD3E nanobody effectively suppressed angiogenesis and tumor cell proliferation in a breast cancer mouse model.^[59]^

In summary, the TME of breast cancer is affected by many factors, such as race, tumor type, clinical stage and molecular subtype.^[31]^ In this study, the correlation between immune/stromal scores and clinical characteristics was analyzed, and the hub genes in the TME of breast IDC were identified. Although similar results have been shown in other studies, they did not focus on breast IDC, and the identified genes were not further studied for their prognostic and clinical significance.^[33,40]^ In addition, previous studies were mainly limited to one database while our study combined two databases to minimize the bias of data.^[34,35,77]^ Nevertheless, more studies can be expected in the future, such as the analysis of infiltrated immune cells in the TME of breast IDC, and the verification of functions and molecular mechanisms of these hub genes *in vitro* and *in vivo*. We hope that our work will provide some new possibilities for the diagnosis and treatment of breast cancer.

## Acknowledgments

We thank International Science Editing (http://www.internationalscienceediting.com) for editing this manuscript.

## Author contributions

**Conceptualization:** Qiang He.

**Data curation:** Qiang He.

**Formal analysis:** Qiang He.

**Investigation:** Qiang He.

**Methodology:** Qiang He.

**Project administration:** Qiang He.

**Resources:** Shuyin Xue.

**Software:** Qiang He.

**Supervision:** Mei He.

**Validation:** Qingbiao Wa, Xinrong Luo.

**Visualization:** Wei Chen.

**Writing – original draft:** Qiang He, Shuang Feng.

**Writing – review & editing:** Zhibing Chen, Xinrong Luo.

## References

[1] Siegel RL Miller KD Jemal A. Cancer statistics, 2020. CA Cancer J Clin 2020;70:07–30. 10.3322/caac.21590 31912902

[2] Kozłowski J Kozłowska A Kocki J. Breast cancer metastasis - insight into selected molecular mechanisms of the phenomenon. Postepy Hig Med Dosw (Online) 2015;69:447–51. 25897105 10.5604/17322693.1148710

[3] Velaei K Samadi N Barazvan B. Tumor microenvironment-mediated chemoresistance in breast cancer. Breast 2016;30:92–100. 27668856 10.1016/j.breast.2016.09.002

[4] Whiteside T. The tumor microenvironment and its role in promoting tumor growth. Oncogene 2008;27:5904–12. 18836471 10.1038/onc.2008.271 PMC3689267

[5] Hanahan D Coussens LM. Accessories to the crime: functions of cells recruited to the tumor microenvironment. Cancer Cell 2012;21:309–22. 22439926 10.1016/j.ccr.2012.02.022

[6] Denton AE Roberts EW Fearon DT. Stromal cells in the tumor microenvironment. Adv Exp Med Biol 2018;1060:99–114. 30155624 10.1007/978-3-319-78127-3_6

[7] Finak G Bertos N Pepin F. Stromal gene expression predicts clinical outcome in breast cancer. Nat Med 2008;14:518–27. 18438415 10.1038/nm1764

[8] Chang HY Nuyten DS Sneddon JB. Robustness, scalability, and integration of a wound-response gene expression signature in predicting breast cancer survival. Proc Natl Acad Sci U S A 2005;102:3738–43. 15701700 10.1073/pnas.0409462102 PMC548329

[9] Farmer P Bonnefoi H Anderle P. A stroma-related gene signature predicts resistance to neoadjuvant chemotherapy in breast cancer. Nat Med 2009;15:68–74. 19122658 10.1038/nm.1908

[10] Nederlof I De Bortoli D Bareche Y. Comprehensive evaluation of methods to assess overall and cell-specific immune infiltrates in breast cancer. Breast Cancer Res 2019;21:151. 31878981 10.1186/s13058-019-1239-4 PMC6933637

[11] Tomioka N Azuma M Ikarashi M. The therapeutic candidate for immune checkpoint inhibitors elucidated by the status of tumor-infiltrating lymphocytes (TILs) and programmed death ligand 1 (PD-L1) expression in triple negative breast cancer (TNBC). Breast Cancer 2018;25:34–42. 28488168 10.1007/s12282-017-0781-0

[12] Lipson EJ Forde PM Hammers HJ. Antagonists of PD-1 and PD-L1 in cancer treatment. Semin Oncol 2015;42:587–600. 26320063 10.1053/j.seminoncol.2015.05.013 PMC4555873

[13] Emens LA Ascierto PA Darcy PK. Cancer immunotherapy: opportunities and challenges in the rapidly evolving clinical landscape. Eur J Cancer 2017;81:116–29. 28623775 10.1016/j.ejca.2017.01.035

[14] Yoshihara K Shahmoradgoli M Martínez E. Inferring tumour purity and stromal and immune cell admixture from expression data. Nat Commun 2013;4:2612. 24113773 10.1038/ncomms3612 PMC3826632

[15] Jia D Li S Li D. Mining TCGA database for genes of prognostic value in glioblastoma microenvironment. Aging (Albany NY) 2018;10:592–605. 29676997 10.18632/aging.101415 PMC5940130

[16] Bai F Jin Y Zhang P. Bioinformatic profiling of prognosis-related genes in the breast cancer immune microenvironment. Aging (Albany NY) 2019;11:9328–47. 31715586 10.18632/aging.102373 PMC6874454

[17] Zhao X Hu D Li J. Database mining of genes of prognostic value for the prostate adenocarcinoma microenvironment using the cancer gene atlas. Biomed Res Int 2020;2020:5019793. 32509861 10.1155/2020/5019793 PMC7251429

[18] Chen CH Lu YS Cheng AL. Disparity in tumor immune microenvironment of breast cancer and prognostic impact: Asian versus Western populations. Oncologist 2020;25:e16–23. 31371522 10.1634/theoncologist.2019-0123 PMC6964121

[19] Ritchie ME Phipson B Wu D. limma powers differential expression analyses for RNA-sequencing and microarray studies. Nucleic Acids Res 2015;43:e47. 25605792 10.1093/nar/gkv007 PMC4402510

[20] Guevara MR Hartmann D Mendoza M. Diverse: an R package to analyze diversity in complex systems. R J 2016;8:60.

[21] Chen H Boutros PC. VennDiagram: a package for the generation of highly-customizable Venn and Euler diagrams in R. BMC Bioinformatics 2011;12:35. 21269502 10.1186/1471-2105-12-35 PMC3041657

[22] Yu G. ClusterProfiler: universal enrichment tool for functional and comparative study. BioRxiv 2018;256784.

[23] Zhang T Jiang M Chen L. Prediction of gene phenotypes based on GO and KEGG pathway enrichment scores. BioMed Res Int 2013;2013:10.1155/2013/870795 PMC3838811 24312912

[24] Shi J Walker MG. Gene set enrichment analysis (GSEA) for interpreting gene expression profiles. Current Bioinform 2007;2:133–7.

[25] Szklarczyk D Franceschini A Wyder S. STRING v10: protein–protein interaction networks, integrated over the tree of life. Nucleic Acids Res 2015;43 (D1):D447–52. 25352553 10.1093/nar/gku1003 PMC4383874

[26] van Dam S Vosa U van der Graaf A. Gene co-expression analysis for functional classification and gene–disease predictions. Brief Bioinform 2018;19:575–92. 28077403 10.1093/bib/bbw139 PMC6054162

[27] Heagerty PJ Saha-Chaudhuri P Saha-Chaudhuri MP. Package ‘survivalROC’ 2013;https://cran.r-project.org/web/packages/survivalROC/survivalROC.pdf

[28] Hoon Tan P Ellis I Allison K. The 2019 WHO classification of tumours of the breast. Histopathology 2020;77:181–5. 32056259 10.1111/his.14091

[29] Pelizzo G Veschi V Mantelli M. Microenvironment in neuroblastoma: isolation and characterization of tumor-derived mesenchymal stromal cells. BMC Cancer 2018;18:1176. 30482160 10.1186/s12885-018-5082-2 PMC6260687

[30] Milioli HH Vimieiro R Tishchenko I. Iteratively refining breast cancer intrinsic subtypes in the METABRIC dataset. BioData Min 2016;9:02. 10.1186/s13040-015-0078-9 PMC4712506 26770261

[31] Mao Y Keller ET Garfield DH. Stromal cells in tumor microenvironment and breast cancer. Cancer Metastasis Rev 2013;32:303–15. 23114846 10.1007/s10555-012-9415-3 PMC4432936

[32] Vincent KM Findlay SD Postovit LM. Assessing breast cancer cell lines as tumour models by comparison of mRNA expression profiles. Breast Cancer Res 2015;17:114. 26289960 10.1186/s13058-015-0613-0 PMC4545915

[33] Li W Sang M Hao X. Gene expression and DNA methylation analyses suggest that immune process-related ADCY6 is a prognostic factor of luminal-like breast cancer. J Cell Biochem 2020;121:3537–46. 31886586 10.1002/jcb.29633

[34] Ren H Hu D Mao Y. Identification of Genes with Prognostic Value in the Breast Cancer Microenvironment Using Bioinformatics Analysis. Med Sci Monit 2020;26:e920212. 32251269 10.12659/MSM.920212 PMC7160604

[35] Li B Geng R Wu Q. Alterations in immune-related genes as potential marker of prognosis in breast cancer. Front Oncol 2020;10:333. 32226776 10.3389/fonc.2020.00333 PMC7080956

[36] Du T Zhu L Levine KM. Invasive lobular and ductal breast carcinoma differ in immune response, protein translation efficiency and metabolism. Sci Rep 2018;8:7205. 29739984 10.1038/s41598-018-25357-0 PMC5940770

[37] Lee AH Happerfield LC Millis RR. Inflammatory infiltrate in invasive lobular and ductal carcinoma of the breast. Br J Cancer 1996;74:796–801. 8795584 10.1038/bjc.1996.438 PMC2074701

[38] Zhao H Langerød A Ji Y. Different gene expression patterns in invasive lobular and ductal carcinomas of the breast. Mol Biol Cell 2004;15:2523–36. 15034139 10.1091/mbc.E03-11-0786 PMC420079

[39] Desmedt C Salgado R Fornili M. Immune infiltration in invasive lobular breast cancer. J Natl Cancer Inst 2018;110:768–76. 29471435 10.1093/jnci/djx268 PMC6037125

[40] Yang H Zhao K Kang H. Exploring immune-related genes with prognostic value in microenvironment of breast cancer from TCGA database. Medicine (Baltimore) 2020;99:e19561. 32243373 10.1097/MD.0000000000019561 PMC7220520

[41] Lakschevitz FS Hassanpour S Rubin A. Identification of neutrophil surface marker changes in health and inflammation using high-throughput screening flow cytometry. Exp Cell Res 2016;342:200–9. 26970376 10.1016/j.yexcr.2016.03.007

[42] Brummer G Fang W Smart C. CCR2 signaling in breast carcinoma cells promotes tumor growth and invasion by promoting CCL2 and suppressing CD154 effects on the angiogenic and immune microenvironments. Oncogene 2020;39:2275–89. 31827233 10.1038/s41388-019-1141-7 PMC7071973

[43] Tong AW Papayoti MH Netto G. Growth-inhibitory effects of CD40 ligand (CD154) and its endogenous expression in human breast cancer. Clin Cancer Res 2001;7:691–703. 11297266

[44] Gomes EM Rodrigues MS Phadke AP. Antitumor activity of an oncolytic adenoviral-CD40 ligand (CD154) transgene construct in human breast cancer cells. Clin Cancer Res 2009;15:1317–25. 19228733 10.1158/1078-0432.CCR-08-1360

[45] Pan W Gong J Yang C. Peripheral blood CD40-CD40L expression in human breast cancer. Irish journal of medical science 2013;182:719–21. 23456134 10.1007/s11845-013-0931-0

[46] Voorzanger-Rousselot N Alberti L Blay J-Y. CD40L induces multidrug resistance to apoptosis in breast carcinoma and lymphoma cells through caspase independent and dependent pathways. BMC Cancer 2006;6:75. 16545138 10.1186/1471-2407-6-75 PMC1435764

[47] Kim H Kim Y Bae S. Direct interaction of CD40 on tumor cells with CD40L on T cells increases the proliferation of tumor cells by enhancing TGF-β production and Th17 differentiation. PLoS One 2015;10:e0125742. 25992978 10.1371/journal.pone.0125742 PMC4436336

[48] Voorzanger-Rousselot N Blay J-Y. Coexpression of CD40 and CD40L on B lymphoma and carcinoma cells: an autocrine anti-apoptotic role. Leukemia & lymphoma 2004;45:1239–45. 15360007 10.1080/1042819032000159834

[49] Wang Q Yang M Zhang Y. Novel Combination Oncolytic Adenoviral Gene Therapy Armed with Dm-dNK and CD40L for Breast Cancer. Current gene therapy 2019;19:54–65. 30848201 10.2174/1566523219666190307094713

[50] Yu J Zhang W Jiang H. CD4+ T cells in CIKs (CD4+ CIKs) reversed resistance to Fas-mediated apoptosis through CD40/CD40L ligation rather than IFN-γ stimulation. Cancer biotherapy & radiopharmaceuticals 2008;23:342–54. 18593367 10.1089/cbr.2007.0454 PMC2936940

[51] Shousha S Costello C Luqmani Y. CD5 positive breast carcinoma in a patient with untreated chronic lymphocytic leukaemia: molecular studies of chromosome 13q. J Clin Pathol 1998;51:862–4. 10193332 10.1136/jcp.51.11.862 PMC500985

[52] Walsh R Peston D Shousha S. Comparison of immunoperoxidase staining of 3 different types of CD5 antibodies in a spectrum of breast lesions. Arch Pathol Lab Med 2001;125:781–4. 11371230 10.5858/2001-125-0781-COISOD

[53] Alotaibi F Rytelewski M Figueredo R. CD5 blockade enhances ex vivo CD8+ T cell activation and tumour cell cytotoxicity. Eur J Immunol 2020;50:695–704. 31943150 10.1002/eji.201948309

[54] Xu F Li D Zhang Q. Association of CD27 and CD70 gene polymorphisms with risk of sporadic breast cancer in Chinese women in Heilongjiang Province. Breast Cancer Res Treat 2012;133:1105–13. 22399187 10.1007/s10549-012-1987-7

[55] Han Y Xie W Song D-G. Control of triple-negative breast cancer using ex vivo self-enriched, costimulated NKG2D CAR T cells. J Hematol Oncol 2018;11:92. 29980239 10.1186/s13045-018-0635-z PMC6035420

[56] Wang J Liu Y Yang Z. CD52 is a prognostic biomarker and correlated with immune features in breast cancer 2020;https://www.researchsquare.com/article/rs-31586/v1

[57] Wang J Zhang G Sui Y. CD52 Is a Prognostic Biomarker and Associated with Tumor Microenvironment in Breast Cancer. Front Genet 2020;11:1350. 10.3389/fgene.2020.578002 PMC7667128 33240323

[58] Moradi-Kalbolandi S Sharifi-K A Darvishi B. Evaluation the potential of recombinant anti-CD3 nanobody on immunomodulatory function. Mol Immunol 2020;118:174–81. 31884389 10.1016/j.molimm.2019.12.017

[59] Khatibi AS Roodbari NH Majidzade AK. In vivo tumor-suppressing and anti-angiogenic activities of a recombinant anti-CD3ε nanobody in breast cancer mice model. Immunotherapy 2019;11:1555–67. 31865872 10.2217/imt-2019-0068

[60] Elgueta R Benson MJ de Vries VC. Molecular mechanism and function of CD40/CD40L engagement in the immune system. Immunol Rev 2009;229:152–72. 19426221 10.1111/j.1600-065X.2009.00782.x PMC3826168

[61] Shahrokhi S Daneshmandi S Menaa F. Tumor necrosis factor-α/CD40 ligand-engineered mesenchymal stem cells greatly enhanced the antitumor immune response and lifespan in mice. Hum Gene Ther 2014;25:240–53. 24372569 10.1089/hum.2013.193 PMC3955978

[62] Marigo I Zilio S Desantis G. T cell cancer therapy requires CD40-CD40L activation of tumor necrosis factor and inducible nitric-oxide-synthase-producing dendritic cells. Cancer Cell 2016;30:377–90. 27622331 10.1016/j.ccell.2016.08.004 PMC5023283

[63] Soliman H Mediavilla-Varela M Antonia SJ. A GM-CSF and CD40L bystander vaccine is effective in a murine breast cancer model. Breast Cancer (Dove Med Press) 2015;7:389–97. 26719725 10.2147/BCTT.S89563 PMC4687618

[64] Chen Y Peubez C Jayne S. Differential activation of pro-survival pathways in response to CD40LG/IL4 stimulation in chronic lymphocytic leukaemia cells. Br J Haematol 2019;184:867–9. 29676456 10.1111/bjh.15197

[65] Vogler M Butterworth M Majid A. Concurrent up-regulation of BCL-XL and BCL2A1 induces approximately 1000-fold resistance to ABT-737 in chronic lymphocytic leukemia. Blood 2009;113:4403–13. 19008458 10.1182/blood-2008-08-173310

[66] Bamberger M Santos AM Gonçalves CM. A new pathway of CD5 glycoprotein-mediated T cell inhibition dependent on inhibitory phosphorylation of Fyn kinase. J Biol Chem 2011;286:30324–36. 21757751 10.1074/jbc.M111.230102 PMC3162391

[67] Alotaibi F Rytelewski M Figueredo R. CD5 blockade enhances ex vivo CD8(+) T cell activation and tumour cell cytotoxicity. Eur J Immunol 2020;50:695–704. 31943150 10.1002/eji.201948309

[68] Hosaka N Ohe C Miyasaka C. The role of CD5 expression in thymic carcinoma: possible mechanism for interaction with CD5+ lymphoid stroma (microenvironment). Histopathology 2016;68:450–5. 26018940 10.1111/his.12742

[69] Das S Bar-Sagi D. BTK signaling drives CD1d(hi)CD5(+) regulatory B-cell differentiation to promote pancreatic carcinogenesis. Oncogene 2019;38:3316–24. 30635655 10.1038/s41388-018-0668-3 PMC6486434

[70] Prasad K Ao Z Yoon Y. CD27, a member of the tumor necrosis factor receptor family, induces apoptosis and binds to Siva, a proapoptotic protein. Proc Natl Acad Sci U S A 1997;94:6346–51. 9177220 10.1073/pnas.94.12.6346 PMC21052

[71] Wajant H. Therapeutic targeting of CD70 and CD27. Expert Opin Ther Targets 2016;20:959–73. 26914723 10.1517/14728222.2016.1158812

[72] Roberts DJ Franklin NA Kingeter LM. Control of established melanoma by CD27 stimulation is associated with enhanced effector function and persistence, and reduced PD-1 expression of tumor infiltrating CD8(+) T cells. J Immunother 2010;33:769–79. 20842060 10.1097/CJI.0b013e3181ee238f PMC2955154

[73] Burris HA Infante JR Ansell SM. Safety and activity of varlilumab, a novel and first-in-class agonist anti-CD27 Antibody, in patients with advanced solid tumors. J Clin Oncol 2017;35:2028–36. 28463630 10.1200/JCO.2016.70.1508

[74] Zhao Y Su H Shen X. The immunological function of CD52 and its targeting in organ transplantation. Inflamm Res 2017;66:571–8. 28283679 10.1007/s00011-017-1032-8

[75] Craig JW Mina MJ Crombie JL. Assessment of CD52 expression in “double-hit” and “double-expressor” lymphomas: Implications for clinical trial eligibility. PLoS One 2018;13:e0199708. 30020951 10.1371/journal.pone.0199708 PMC6051601

[76] Salmerón A Sánchez-Madrid F Ursa MA. A conformational epitope expressed upon association of CD3-epsilon with either CD3-delta or CD3-gamma is the main target for recognition by anti-CD3 monoclonal antibodies. J Immunol 1991;147:3047–52. 1717585

[77] Xu M Li Y Li W. Immune and Stroma Related Genes in Breast Cancer: A Comprehensive Analysis of Tumor Microenvironment Based on the Cancer Genome Atlas (TCGA) Database. Front Med (Lausanne) 2020;7:64. 32195260 10.3389/fmed.2020.00064 PMC7066229

